# Consulting r/Transplant: Assessment of Reddit Use and Sentiment in Solid Organ Transplantation

**DOI:** 10.1097/TXD.0000000000001922

**Published:** 2026-02-23

**Authors:** Ava Herzog, Divya Goyal, Flavio Paterno, Arpit Amin, James V. Guarrera, Keri E. Lunsford, Grace S. Lee-Riddle

**Affiliations:** 1 Albany Medical College, Albany, NY.; 2 Rensselaer Polytechnic Institute, Troy, NY.; 3 Division of Transplant and HPB Surgery, Department of Surgery, Rutgers New Jersey Medical School, Newark, NJ.

## Abstract

**Background.:**

Solid organ transplants are life-changing experiences that impact recipients, donors, care partners, medical teams, communities, and popular media. Social media is an influential part of modern life and provides a platform for healthcare experiences. This study assessed perspectives on solid organ transplantation by exploring the transplant community’s utilization of the social media site Reddit.

**Methods.:**

The 1000 most recent and “Top” 100 posts from the r/transplant subreddit were extracted in April 2024. Posts underwent categorization to identify key themes and subthemes. The Python “Valence Aware Dictionary and sEntiment Reasoner” sentiment analysis tool was used to evaluate post sentiment.

**Results.:**

Posts most frequently discussed kidney (43.3%) and liver (18.3%) transplants. Most were authored by transplant recipients (66.5%) and centered on the posttransplant period (63.0%). Categorization of the 1000 most recent posts revealed 11 major themes: eligibility, pretransplant anticipation, logistics, early recovery, late recovery, medication, lifestyle, infection, seeking connection, sharing experience, and transplant in media. Over a third of posts sought Reddit user input on medical questions. Sentiment analysis demonstrated predominantly positive sentiment (0.25). The “Top” 100 posts had more positive sentiment (0.50) and focused on sharing experiences.

**Conclusions.:**

Reddit serves as a platform for information exchange and support for the transplant community, addressing a wide range of pre- and posttransplant topics. Future work should explore ways to incorporate the real-world solutions offered by social media into formal healthcare practices.

## INTRODUCTION

Solid organ transplantation provides definitive treatment for end-stage organ dysfunction with increasing numbers of transplants annually.^1^ Although health-related quality of life generally increases after transplant, suboptimal clinical outcomes or difficult recoveries can affect individual experiences.^2^ These life-changing experiences affect not just transplant recipients but also their support systems.^3,4^ Living and deceased donors, along with their families and care partners, multidisciplinary medical teams, communities, government agencies, and popular media all play a significant role and are impacted by the transplant process.

Social media is integrated into everyday life and can be a source of information, misinformation, community, and support.^5,6^ Reddit is a social media platform that is “home to thousands of communities, endless conversation, and authentic human connection” via anonymous posts, votes, and comments within topic-specific discussion forums called “subreddits.”^7^ In 2024, Reddit had >97 million active users, establishing Reddit as the third most visited website in the United States based on traffic metrics.^8,9^

Reddit forums addressing healthcare topics facilitate sharing anxieties, preparing for appointments, obtaining symptom validation, or identifying potential diagnoses.^10-12^ Reddit users also note discrepancies between physician expectations and patients’ real-life experiences with notable frustration.^11^ Although Reddit can potentially enhance health education for the general public, it is also a potential source of inaccurate medical information.^13^

Prior work on social media and transplant examined the use of individual social media platforms to identify living donors, increase organ donation, and advertise transplant centers.^14,15^ Previous studies did not include Reddit, and there is limited understanding of transplant-related content available on this platform. The solid organ transplant subreddit (r/transplant) is a large active community within Reddit’s medical communities with 14 000 members, >13 000 weekly visitors, and >800 contributions weekly.^16^ As the use of various social media platforms increases, transplant providers should understand the range of information available to the transplant community online. This study aimed to (1) analyze how the transplant community utilizes Reddit and (2) gauge Reddit users’ sentiments on undergoing solid organ transplant.

## MATERIALS AND METHODS

### Data Source and Collection

On Reddit’s r/transplant subreddit site, the 1000 most recent posts were extracted using a Reddit application programming interface in April 2024. We did not include posts in other transplant-related subreddits such as r/kidneytransplant, r/lungtranspant, or r/dialysis. Posts from r/transplant were filtered by “New.” Post titles, post content, and the “Best” two comments, as ranked by upvotes and downvotes, were recorded. Comments were utilized to provide context for the primary post’s title and content but were not analyzed. The “Top” 100 r/transplant posts with the greatest upvote-to-downvote ratio were separately extracted and filtered by “Top.”

### Data Analysis

Extracted posts were exported to Microsoft Excel and underwent categorization between 2 authors (D.G. and G.S.L.-R.). D.G. is a female undergraduate student without background in transplant and G.S.L.-R. is a female transplant surgeon (MD, MSME) with expertise in medical ethics and experience with qualitative research. Both served as independent coders. A theme key was iteratively developed through an inductive approach where posts were independently reviewed in sets of 100 by each author for major themes and reconciled until theme saturation was achieved and a theme key was established. The remaining posts were independently coded according to the theme key, and the results were then reviewed and reconciled between the 2 authors. Posts that represented multiple themes were reviewed by both authors and the authors jointly decided on 1 theme the post represented more clearly for categorization. Posts were then divided into major themes, and subthemes were identified within each major theme. Major themes and subthemes were reviewed with all authors to ensure clarity and internal validity. The theme key was then applied to the “Top” 100 r/transplant posts. The study adhered to the consolidated criteria for reporting qualitative research guidelines (**Table S1, SDC**, https://links.lww.com/TXD/A838).

Data were also extracted from post content to determine demographic information (organ type, relation to transplant, time from transplant, pediatric transplant, and outside US). Post authors were categorized as transplant recipient, living donor, primary caretaker (primary care partner for a patient), family member (family member who identified as not the primary care partner), medical professional, or other. Time from transplant was categorized as pretransplant, posttransplant, or unknown. The posttransplant timeframe was further subdivided as <3 mo, 3–12 mo, 1–10 y, >10 y, or unspecified.

Sentiment analysis was performed via Valence Aware Dictionary for sEntiment Reasoning (VADER), a natural language processing Python tool that is publicly available and previously validated in biomedical literature for sentiment analysis.^17-19^ VADER analyzes social media text to generate a compound score for each post, providing a normalized value ranging from −1 as the most negative and +1 as the most positive. The overall sentiment rating of each post was determined using the following compound score cutoffs: negative (≤−0.05), neutral (>−0.05 to <0.05), or positive (≥0.05), as previously validated.^10^

Nonparametric testing was performed with Kruskal-Wallis and Mann-Whitney H tests to determine statistical significance. A *P* value of <0.05 was considered statistically significant.

### Ethics Approval

This study was considered exempt by the Rutgers University Institutional Review Board (Pro2024000692).

## RESULTS

### Recent 1000 r/Transplant Posts Content

Demographic information for recent 1000 r/transplant posts is shown in Table 1. The most frequently discussed solid organ transplant types were kidney (43.3%) and liver (18.3%) transplants. Most posts were written by transplant recipients (66.5%), followed by primary caretakers (8.3%), family members (8.3%), and living donors (6.9%). Only 3 posts were written by medical professionals. Most posts focused on the posttransplant period (63.0%). Few posts specifically mentioned pediatric transplant (1.4%) or transplant experience outside of the United States (2.7%).

**TABLE 1.** Demographics of most recent 1000 r/transplant posts

	N (%)
Organ type	
Kidney	433 (43.3)
Liver	183 (18.3)
Heart	96 (9.6)
Lung	64 (6.4)
Multiorgan	43 (4.3)
Pancreas	8 (0.8)
Intestine/multivisceral	2 (0.2)
Not specified	171 (17.1)
Relation to transplant	
Living donor	69 (6.9)
Recipient	665 (66.5)
Primary caretaker	83 (8.3)
Family member	83 (8.3)
Medical professional	3 (0.3)
Other	97 (9.7)
Time from transplant	
Pretransplant	305 (30.5)
Posttransplant	630 (63.0)
<3 mo	160 (16.0)
3–12 mo	91 (9.1)
1–10 y	147 (14.7)
>10 y	40 (4.0)
Not specified	192 (19.2)
Unknown	65 (6.5)
Pediatrics	14 (1.4)
International (outside United States)	27 (2.7)

**FIGURE 1.**
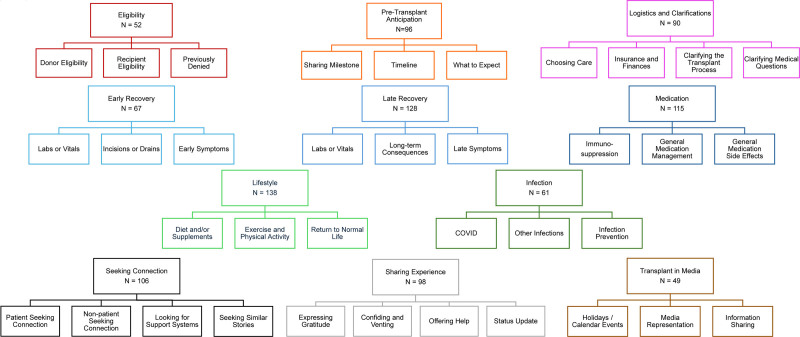
Major themes and subthemes.

Categorization of the recent 1000 r/transplant posts revealed eleven major themes, along with additional subthemes, shown in Figure 1. Table 2 displays each major theme with representative quotes. Table 3 displays each major theme, subtheme, and representative quotes for each subtheme.

**TABLE 2.** Representative quotes from each major theme

Major theme	Representative quote
Eligibility	The medical ethics Dr. […] on the radio […] said that disabled people could not get on the transplant list. This really stuck with me because my adult son is disabled and has some minor liver issues. Is this really true? Is it because they won’t be able to care for themselves after a transplant? Or because there are other more healthy people that could use the transplant more? Any insight on this would be helpful for me to understand more on this issue.
Pretransplant anticipation	I’m already in the hospital sick. Got the call for new lungs a couple hours ago. I am extremely nervous. Still didn’t think it would happen for a bit. Hope I’m up for this!
Logistics and clarifications	For those of you lucky enough to have a choice, how did you choose your transplant center? What factors in retrospect were not so important, or which ones would you have paid more attention to? I’m in the Chicago area and there a quite a few to choose from. Of course volume, experienced team, etc but maybe there are other things to consider?
Early recovery (<3 mo posttransplant)	Labs are slowly getting better, creatinine was 11 before surgery and 5 last night. But hemoglobin is going down […]. It was 11 before surgery and 8.8 this morning. Anyone else have their hemoglobin fall right after surgery and recover soon after? I’m hoping I’m not bleeding internally. I just checked the labs on the app, and have yet to talk to someone about it.
Late recovery (>3 mo posttransplant)	Has anyone on here received a second double lung TX? I’ve had my first transplant 12 years and now after a few years of chronic rejection my lung function is bottomed out at 20% on and off oxygen. Hoping to be considered for another transplant just wondering if anyone has experienced the same or even had multiple transplants.
Medication	How often do you take pills after transplant? Everyday? Twice daily? Every 8 hours? I’m looking at pill organizers and just trying to see what would be best. The one I have now I can’t even fit all my meds in for the day inside.
Lifestyle	How long after surgery until you’re able to go swimming? It’s one of the biggest things I miss is swimming with my daughter.
Infection	I did everything I should be doing but I have come down with COVID for the first time. […] I thought it was a cold but saw that I was getting a low grade fever (below 100), I’m glad I checked. Just took my first does of Paxlovid this morning.
Seeking connection	My dad has been on the list for 8 months now. Had two dry runs so far, one happening literally last Sunday. He’s taken a really bad turn and he’s in the hospital due to his breathing. […] I’m trying so hard to be positive but I’m so scared. Does anyone have any advice? I’ve been crying on and off since yesterday and can’t seem to stop. I only ever see stories about how the transplant doesn’t work or it’s too late.
Sharing experience	Hey guys I posted on here a while ago and finally ended up getting my heart transplant. I […] started living in the ICU February 5th and had the transplant on March 21st! I had my chest tubes taken today (just 2). And already have some iv meds I’ve been taken off.
Transplant in media	Did anyone catch the latest Last Week Tonight? John Oliver did a segment on organ donation! I thought it was really well done, I wasn’t aware of all the issues with OPOs and I learned a lot. I definitely teared up at the end, knowing that lots of people don’t make it in time for a transplant definitely makes me feel survivors guilt.

**TABLE 3.** Representative quotes from each subtheme

Major theme	Subthemes	Representative quote
Eligibility	Donor eligibility	I’m trying to donate my kidney to my wife. She is AB and I am O+. Has anyone had experience with this type of donation.
	Recipient eligibility	The medical ethics Dr. […] on the radio […] said that disabled people could not get on the transplant list. This really stuck with me because my adult son is disabled and has some minor liver issues. Is this really true? Is it because they won’t be able to care for themselves after a transplant? Or because there are other more healthy people that could use the transplant more? Any insight on this would be helpful for me to understand more on this issue.
	Previously denied	Hey, getting second heart transplant at 70. Most places don’t do at 70, so my place referred me to another place that might. Went through 3-4 months of waiting and testing. Heart team said yes, kidney team said no. I guess need a kidney for later down the road. […] Now getting referred to two more places. […] I want my name out there so [every] hospital has the option to take me, I don’t care how far I have to travel.
Pretransplant anticipation	Sharing milestone	I’m already in the hospital sick. Got the call for new lungs a couple hours ago. I am extremely nervous. Still didn’t think it would happen for a bit. Hope I’m up for this!
	Timeline	I’m currently waiting for an SPK transplant and I was told the wait is about 1 1/2 years for my blood type. Soon after I got a new transplant coordinator and he told me the wait is 4-5 years. […] I hate calling calling all the time and I really don’t want them to be annoyed but I also would like a straight answer.
	What to expect	I am newly active on the transplant list and am wondering what I should have packed and ready to go when the phone rings.
Logistics and clarifications	Choosing care	For those of you lucky enough to have a choice, how did you choose your transplant center? What factors in retrospect were not so important, or which ones would you have paid more attention to? I’m in the Chicago area and there a quite a few to choose from. Of course volume, experienced team, etc but maybe there are other things to consider?
	Insurance and finances	Hello! Its that dreaded time (at least for US-based folks). I am trying to decide between BCBS or UHC Choice Gold plan. My gut is leaning with Blue Cross as I’ve had it for my transplant last year and do not want to rock the boat. But I think It’d be a good opportunity to discuss what were electing this year and why. To spread knowledge of what to look for in plans etc. Things I look for is cost of labs, imaging, ER visit, prescriptions (tiers), hospital admission, out-of-state coverage, and over-all deductible.
	Clarifying the transplant process	Writing a thank you letter to a deceased donor’s family for their gift is difficult. For those who have received a cadaver organ(s), did you write to the donor’s family, if so how long after your transplant did you write it and did you hear back from the family?
	Clarifying medical questions	Can anyone please explain to me how cpra are calculated? and what’s considered a good number Thanks.
Early recovery	Labs or vitals	Labs are slowly getting better, creatinine was 11 before surgery and 5 last night. But hemoglobin is going down […]. It was 11 before surgery and 8.8 this morning. Anyone else have their hemoglobin fall right after surgery and recover soon after? I’m hoping I’m not bleeding internally. I just checked the labs on the app, and have yet to talk to someone about it.
	Incisions or drains	I am 2 weeks posttransplant and my team said I can start scar care in a week or 2. What did you do to make the scar look nicer?
	Early symptoms	Everyone talks about how you instantly feel better after a kidney transplant, but I honestly kinda feel worse. I can barely walk up the stairs without feeling overtired. I’m 3 weeks post op so I know it’s soon, Im just so sick of feeling sick.
Late recovery	Labs or vitals	My husband is two years out from a DD kidney transplant. His creatinine has jumped from 1.5 (baseline is 1.4-1.6) to 1.9. He is very upset. We called the transplant clinic and they said they’ll talk to the doctor about it. All his other numbers look normal for him. Any advice to calm my husband’s concerns? The transplant journey is a rollercoaster, for sure.
	Long-term consequences	Has anyone on here received a second double lung TX? I’ve had my first transplant 12 years and now after a few years of chronic rejection my lung function is bottomed out at 20% on and off oxygen. Hoping to be considered for another transplant just wondering if anyone has experienced the same or even had multiple transplants.
	Late symptoms	Was wondering if anyone else is dealing with stubborn and severe back and neck acne? For context, I (30M) had a liver transplant about 8 years ago and taking Prograf 2.5mg twice a day. A year after my transplant, I got pretty severe neck and back acne that wasn’t able to be permanently treated with antibiotics (doxycycline and azithromycin). […] My dermatologist also doesn’t know what else there is to do besides accutane which is not recommended to take. Just wondering if anyone else is in a similar situation or had acne and was able to treat it?
Medication	Immunosuppression	They told me shakes are common if my tacro levels are too high, but my last blood work came back fine. But my hands won’t stop shaking. Like to the point where I can hardly cut my steak. I’m 3 weeks post op. Is there something else that can be causing this?
	General medication management	How often do you take pills after transplant? Everyday? Twice daily? Every 8 hours? I’m looking at pill organizers and just trying to see what would be best. The one I have now I can’t even fit all my meds in for the day inside.
	General medication side effects	With the dozen+ medications after transplant, does anyone have any success managing the ringer it all puts on your gut? I’m struggling daily with bad cramps, bloating, constipation. […] I feel like I’m trying everything
Lifestyle	Diet and/or supplements	I need you all to please guide and advice me what [the] right diet should be the next 3 months. I’ve heard stories of weight gain, bloating, diabetes, food infections [like Acinetobacter] […] entering the lungs because [of] food contamination. Your experience and expertise will go a long way. Please do share.
	Exercise and physical activity	How long after surgery until you’re able to go swimming? It’s one of the biggest things I miss is swimming with my daughter.
	Return to normal life	So I was on dialysis for 9 years then recently had a kidney transplant 2 months ago. My work history stopped 9 years ago. Those jobs were strenuous, so much heavy lifting. Now I can’t do those anymore. Any examples I can apply for starting from zero experience.
Infection	COVID	I did everything I should be doing but I have come down with COVID for the first time. […] I thought it was a cold but saw that I was getting a low grade fever (below 100), I’m glad I checked. Just took my first does of Paxlovid this morning.
	Other infections	We are now inpatient at transplant hospital for at least a week or more. They have him on IV gancyclovir twice a day and will test CMV every three days. He is also IVIG and they completely stopped his tacro and lowered his cellcept. CMV is no joke! I wonder if he will be on Valcyte for life? Anyone else go through this.
	Infection prevention	How do you deal with your kid bringing home sickness when you’ve got a transplant to manage? Our kid is starting in daycare next year and I’m pretty worried about the impact it might have on my partner, who is 8 years post SPK (pancreas/kidney) transplant.
Seeking connection	Patient seeking connection	I don’t think I need a therapist to work through these feelings, but I would appreciate any advice on introspection and reconciling going from relatively well to unhealthy back to whatever the end result might be after transplant.
	Nonpatient seeking connection	My dad has been on the list for 8 months now. Had two dry runs so far, one happening literally last Sunday. He’s taken a really bad turn and he’s in the hospital due to his breathing. […] I’m trying so hard to be positive but I’m so scared. Does anyone have any advice? I’ve been crying on and off since yesterday and can’t seem to stop. I only ever see stories about how the transplant doesn’t work or it’s too late.
	Looking for support systems	Hi! i’m 22F currently on the waiting list for a kidney+liver transplant. Currently on hemodialysis for my kidney and have very mild but present liver symptoms as well. Looking for other younger people going through the same thing if anyone wants to be friends? that would be awesome:) Also want to ask what has helped you cope with your disease? For me it’s trying to live as normal of a life as possible with my limitations and exercising twice a week. As well as journaling. Sending love to everyone in this subreddit
	Seeking similar stories	My best friend (40f) was diagnosed with severe portal vein hypertension (full blockage) and is having a shunt put in. She was told that this is typically done before a transplant but that for her it will hopefully stave off the need for a transplant (her liver is relatively healthy.) I’m just curious to hear about any experiences with this procedure, recovery, outcome etc
Sharing experience	Expressing gratitude	I received a Liver Transplant in April 2023. I was told today that ***ALL*** my blood work is within normal range and i am currently healthy. I am no longer anemic, yellow, bloated, black and blue, etc! I feel great and cant wait to live the rest of my life happy and healthy!
	Confiding and venting	I donated one of my kidneys almost 13 years ago to a stranger at the time, who became a good friend. Our transplant anniversary would have been on Valentine’s Day. His death wasn’t related to anything with the transplant, so my kidney did its job all the way to the end. I’m feeling grief for my friend and his sudden passing, and a surprising emptiness inside I wasn’t expecting. I think a part of me just thought he would always live and we would always have our bond. It feels very lonely in a way I can’t describe. I didn’t know where to share this feeling in a place that would resonate.
	Offering help	Writing this post to update and to share hope for anyone out there currently dealing with the complications of their loved ones posttransplant. They are resilient and they will get through it! Some just need a bit of extra time and of course please reach out if you need any extra support. This forum helped me greatly when I needed it the most and I’d like to give back.
	Status update	Hey guys I posted on here a while ago and finally ended up getting my heart transplant. I […] started living in the ICU February 5th and had the transplant on March 21st! I had my chest tubes taken today (just 2). And already have some iv meds I’ve been taken off.
Transplant in media	Holidays/calendar events	April is National Donate Life Month. Thank you to all the donors and their families that have given us our gifts.
	Media representation	Did anyone catch the latest Last Week Tonight? John Oliver did a segment on organ donation! I thought it was really well done, I wasn’t aware of all the issues with OPOs and I learned a lot. I definitely teared up at the end, knowing that lots of people don’t make it in time for a transplant definitely makes me feel survivors guilt.
	Information sharing	First human transplant of a genetically modified pig kidney performed

Pretransplant themes include eligibility (donor and recipient eligibilities, previous denial for transplant), pretransplant anticipation (sharing milestones up to transplant, timeline to transplant, what to expect), and logistics (choosing where to obtain care, insurance and financial issues, clarifying the transplant process, clarifying medical questions). Posttransplant themes include early (<3-mo posttransplant) and late (>3-mo posttransplant) recovery (labs or vitals, incisions or drains, posttransplant symptoms, and long-term consequences of transplant), medication (immunosuppression, medication management/adherence, side effects of non-immunosuppressive medications), infection (coronavirus SARS-CoV-2 [COVID], other infections, infection prevention), and lifestyle (diet and/or supplements, exercise and physical activity, return to normal life). Additional major themes include seeking connection (patient or nonpatient seeking connection, looking for support systems, seeking similar stories), sharing experiences (expressing gratitude, confiding and venting, offering help, providing status updates peritransplant), and transplant in media (celebrating holidays or calendar events, media representation of transplant, news and information sharing).

Four additional themes emerged that spanned across major themes: seeking medical advice *despite* engaging their healthcare team, seeking medical advice *without* engaging their healthcare team, hair loss, and marijuana. Table 4 displays these additional themes and representative quotes.

**TABLE 4.** Other themes across major themes and representative quotes

Other themes	N	Representative quote
Seeking Reddit user input alongside professional medical advice	197	Received a kidney transplant on 31 Jan 23, and my Uric acid is over 13. I am having an out flair-up. Saw my doctor today and was put on 40 mg of prednisone on top of my 5 mg of prednisone for a total of 45 mg for 4 days. two questions: Is being 45 mg okay for four days with the new kidney? […] Is uric acid being high indicating there is a problem with the new kidney? […] Thank you Reddit doctors:) I did ask my doctors the same question just want a second opinion. Plus I am impatient and want answers now.
Should consult a medical professional	174	So I had my transplant living donor […] everything has gone great. But I have really bad health anxiety last Tuesday 4 days ago my GFR was 95. However since yesterday my eyes are swollen. I haven’t had any of this since prior to my transplant? Is this normal? Or is this rejection. Let me know if there’s a fix to this.
Hair loss	18	My spouse is about 6 months in post kidney transplant. They have been extremely depressed with their hair constantly falling out because of the Tacrolimus. Biotin doesn’t seem to be doing much. […] any advice on anything that helped with the hair loss?
Marijuana	11	Transplant team said no smoking weed after transplant, but edibles are fine. What about the oil pen vapes? I asked and they had no idea since it’s all still pretty new.

### “Top” 100 r/Transplant Posts Content

Kidney (50.0%), liver (12.0%), and heart (12.0%) transplants were the most upvoted solid organ transplants on Reddit. Most posts were written by transplant recipients (81.0%), followed by living donors (13.0%). The majority of upvoted posts focused on the posttransplant period (87.0%). Sharing experience was the most common major theme (N = 76).

### Sentiment Analysis

The sentiment of the recent 1000 posts was predominantly positive (62.1%) as opposed to negative (30.5%) or neutral (7.4%). The overall VADER score across the 1000 most recent posts was 0.25, denoting a positive sentiment. Major themes with the most positive sentiment were transplant in media (0.53), sharing experience (0.50), pretransplant anticipation (0.47), and seeking connection (0.37). Major themes with the most negative sentiment scores were medication (−0.05), early recovery (0.06), and late recovery (−0.01). Sentiment surrounding hair loss was the most negative theme (−0.16). The sentiment of the “Top” 100 posts was positive (68.0%) as opposed to negative (27.0%) or neutral (5.0%). The average overall VADER score was 0.50, denoting a positive sentiment. Overall sentiment of the “Top” 100 posts was statistically significantly more positive than the 1000 most recent posts (*P* < 0.01).

## DISCUSSION

Reddit is among the most widely utilized websites on the internet and often appears as 1 of the top sources of information for individuals seeking answers online. End-stage organ failure and navigating the road to solid organ transplant can be highly disruptive for patients and their support systems; therefore, it is not surprising that people may turn to the internet for answers. The r/transplant subreddit covers a range of pre- and posttransplant topics and appears to have a mixed purpose: to facilitate information sharing and to provide support. Previous research highlighted the role of social media in campaigning for transplant centers and organ donation, as well as identifying living donors. Our study adds to the literature by characterizing additional purposes of social media through Reddit.

Without ample resources or access to transplant professionals, patients and families may naturally turn to the internet and social media for information. Over a third of posts contained clinical questions that could or should be addressed with a medical professional. Although individual users may choose to add their own disclaimer to their posts, there are no universal medical disclaimers or regulation of content on Reddit. First-person accounts may be more memorable and personable than medical advice, increasing their saliency.^20^ Reddit facilitates users to find similar stories to gather information on common experiences, with authors asking, “Has anyone else experienced…” or “Is this normal?” Transplant professionals are minimally engaged in r/transplant and may thus be unaware of the robust dialogue occurring on the internet. Without transplant professional input, nuanced differences between provider and center practices and policies may be misinterpreted by the public. Information sharing risks the spread of misinformation as well as useful information. Transplant providers should understand and consider how patients may obtain information outside of healthcare settings.

Information-seeking on Reddit alludes to discrepancies between the patient experience and medical care. Hair loss emerged as a theme across the major themes and reflected the most negative sentiment of all themes. Previous literature notes that although hair and other cosmetic changes are common after transplant, providers underestimate the impact of immunosuppression-related physical changes, and treatment is recommended less than half of the time.^21,22^ A lack of symptom validation by healthcare professionals may explain why patients turn to other patients through social media to troubleshoot problematic personal symptoms.

Adequate social and psychological support throughout the transplant process is integral to recovery, both physically and psychologically.^3,4,23,24^ However, time, distance, and transportation access may limit patient and care partner engagement with transplant support groups.^25^ Furthermore, although follow-up with the transplant team is intense and frequent while awaiting transplant and in the immediate posttransplant period, long-term care may be transitioned to local nontransplant providers, creating a gap in access to information and support.

Social media can provide an opportunity for connection, community, and mentorship, particularly for individuals undergoing similar experiences or for those who are socially or geographically isolated.^26,27^ We found providing support and sharing experiences to be considerable functions of r/transplant. Although social support is often a requisite for transplant listing, it is focused on primary care partners and “poor social support” may be cited as a reason for an individual’s transplant denial. Social media has been proposed as an intervention to augment social support to improve access and equity in transplant.^28^ Having diverse support, such as mentors through the r/transplant community, could be protective for patients by providing an outlet for connecting on lived experiences.

With better access to information, patients may be more empowered to participate in healthcare decision-making^29^; however, it is unclear how providers can leverage social media to better facilitate patient care. A survey of the American Society of Transplant Surgeons noted that members utilize social media in their personal and professional lives, but they less frequently utilize social media in their clinical role.^14^ One prior model integrating social media and transplant professionals was Liver Space and Kidney Space, which integrated a health app with Facebook. These apps connected patients and used transplant professionals to screen posted content and provide reliable responses within forums.^28,30^ Future work may address the feasibility of transplant professionals utilizing existing social media platforms to answer specific patient questions and concerns at either an institutional level or through professional organizations.

This study has several limitations. This cross-sectional study of 1000 recent and “Top” 100 posts on r/transplant does not assess the entire repository of r/transplant posts or comments. No data could be collected regarding the transplant center associated with the r/transplant post. The anonymous nature of Reddit limits the ability to obtain certain demographic data such as age, sex, race, rural/urban status; therefore, it is uncertain who the typical r/transplant user is and whether the themes represent the entire spectrum of the transplant community. Analyzed posts were in English, and themes may not be generalizable to populations where English is not the primary language. Unless explicitly stated, Reddit posts were presumed to originate in the United States, thereby limiting the ability to extrapolate our findings to a global context. Reddit is 1 of several social media platforms in use throughout the world and does not represent the entirety of the way the global transplant community interfaces with social media. However, given Reddit’s prominence in internet searches and the lack of prior analysis of the platform, we chose to focus our study on this single platform. Furthermore, social media is heterogenous and performing an analysis across all social media outlets may lose granularity. Finally, while we noted when individuals posted content asking medical questions, we did not a priori plan to assess and analyze the rate of misinformation within posts. This could be a course for future analysis as transplant professionals weigh their engagement with Reddit.

In conclusion, Reddit users in the r/transplant community represent a wide range of transplant patients, support systems, and organ types. Content covers pretransplant topics such as eligibility, anticipation, and logistics, and posttransplant topics such as postoperative recovery, medication, infection, and lifestyle. Reddit users utilize r/transplant for both information and support. Social media platforms such as Reddit have organically filled the needs of the transplant community. Future efforts to improve patient-centered care and communication should consider integrating the real-world solutions already offered by social media platforms.

## ACKNOWLEDGMENTS

The authors would like to acknowledge the users of Reddit’s r/transplant subreddit who are generating interesting and robust dialogue and support for the transplant community.

## Supplementary Material


