# Supplementary material for: Consulting r/Transplant: Assessment of Reddit Use and Sentiment in Solid Organ Transplantation
Source: Transplant Direct. 2026 Feb 23;12(3):e1922. doi: 10.1097/TXD.0000000000001922 (PMC12931940; doi:10.1097/TXD.0000000000001922)
Supplement: Supplementary file 1 [file txd-12-e1922-s001.pdf]

Supplemental Table S1

COREQ (Consolidated criteria for reporting qualitative studies): 32-item checklist

Developed from:
Tong A, Sainsbury P, Craig J. Consolidated criteria for reporting qualitative research (COREQ): a 32-item checklist for interviews and focus groups. International Journal for Quality in Health Care. 2007. Volume 19, Number 6: pp. 349 – 357

Item No	Guide Questions/Description	Reported
**Domain 1: Research team and reflexivity**		
**Personal Characteristics**		
1. Interviewer/ facilitator	Which author/s conducted the interview or focus group?	X
2. Credentials	What were the researcher's credentials? E.g., PhD, MD	X
3. Occupation	What was their occupation at the time of the study?	X
4. Gender	Was the researcher male or female?	X
5. Experience and training	What experience or training did the researcher have?	X
**Relationship with participants**		
6. Relationship established	Was a relationship established prior to study commencement?	N/A
7. Participant knowledge of the interviewer	What did the participants know about the researcher? e.g. personal goals, reasons for doing the research?	N/A
8. Interviewer characteristics	What characteristics were reported about the interviewer/facilitator? e.g. Bias, assumptions, reasons and interests in the research topic	N/A
**Domain 2: study design**		
**Theoretical framework**		
9. Methodological orientation and Theory	What methodological orientation was stated to underpin the study? e.g. grounded theory, discourse analysis, ethnography, phenomenology, content analysis	X
**Participant selection**		
10. Sampling	How were participants selected? e.g., purposive, convenience, consecutive, snowball	X

Item No	Guide Questions/Description	Reported
11. Method of approach	How were participants approached? e.g., face-to-face, telephone, mail, email	N/A
12. Sample size	How many participants were in the study?	N/A
13. Non-participation Setting	How many people refused to participate or dropped out? Reasons?	N/A
14. Setting of data collection	Where was the data collected? e.g., home, clinic, workplace	X
15. Presence of nonparticipants	Was anyone else present besides the participants and researchers?	N/A
16. Description of sample	What are the important characteristics of the sample? e.g. demographic data, date	X
**Data collection able**		
17. Interview guide	Were questions, prompts, and guides provided by the authors? Was it pilot tested?	N/A
18. Repeat interviews	Were repeat interviews carried out? If yes, how many?	N/A
19. Audio/visual recording	Did the research use audio or visual recording to collect the data?	No
20. Field notes	Were field notes made during and/or after the interview or focus group?	N/A
21. Duration	What was the duration of the interviews or focus group?	N/A
22. Data saturation	Was data saturation discussed?	X
23. Transcripts returned	Were transcripts returned to participants for comment and/or correction?	N/A
**Domain 3: analysis and findings**		
**Data analysis**		
24. Number of data coders	How many data coders coded the data?	X
25. Description of the coding tree	Did the authors provide a description of the coding tree?	X
26. Derivation of themes	Were themes identified in advance or derived from the data?	X
27. Software	What software, if applicable, was used to manage the data?	X
28. Participant checking	Did participants provide feedback on the findings?	N/A
**Reporting**		
29. Quotations presented	Were participant quotations presented to illustrate the themes/findings? Was each quotation identified? e.g., participant number	X
30. Data and findings consistent	Was there consistency between the data presented and the findings?	X
31. Clarity of major themes	Were major themes clearly presented in the findings?	X

Item No	Guide Questions/Description	Reported
32. Clarity of minor themes	Is there a description of diverse cases or a discussion of minor themes?	X
